# Growth-Promoting Effect of Cava Lees on Lactic Acid Bacteria Strains: A Potential Revalorization Strategy of a Winery By-Product

**DOI:** 10.3390/foods10071636

**Published:** 2021-07-15

**Authors:** Salvador Hernández-Macias, Oriol Comas-Basté, Anna Jofré, Sara Bover-Cid, M. Luz Latorre-Moratalla, M. Carmen Vidal-Carou

**Affiliations:** 1 Departament de Nutrició, Ciències de l’Alimentació i Gastronomia, Facultat de Farmàcia i Ciències de l’Alimentació, Campus de l’Alimentació de Torribera, Universitat de Barcelona (UB), Av. Prat de la Riba 171, 08921 Santa Coloma de Gramenet, Spain; salva.hernandez@ub.edu (S.H.-M.); oriolcomas@ub.edu (O.C.-B.); mariluzlatorre@ub.edu (M.L.L.-M.); 2 Institut de Recerca en Nutrició i Seguretat Alimentària (INSA·UB), Universitat de Barcelona (UB), Av. Prat de la Riba 171, 08921 Santa Coloma de Gramenet, Spain; 3 Xarxa d’Innovació Alimentària (XIA), C/Baldiri Reixac 4, 08028 Barcelona, Spain; 4 Food Safety and Functionality Programme, Institute of Agrifood Research and Technology (IRTA), Finca Camps i Armet s/n, 17121 Monells, Spain; anna.jofre@irta.cat (A.J.); sara.bovercid@irta.cat (S.B.-C.)

**Keywords:** cava lees, by-product, winery, ß-glucans, mannooligosaccharides (MOS), lactic acid bacteria (LAB), prebiotics, probiotics, revalorization, circular economy

## Abstract

The growing trend of circular economy has prompted the design of novel strategies for the revalorization of food industry by-products. Cava lees, a winery by-product consisting of non-viable cells of *Saccharomyces cerevisiae* rich in β-glucans and mannan-oligosaccharides, can be used as a microbial growth promoter, with potential food safety and health applications. The aim of this study was to assess in vitro the effect of cava lees on the growth of 21 strains of lactic acid bacteria (LAB) species commonly used as starter cultures and/or probiotics. Firstly, 5% of cava lees was selected as the most effective amount for enhancing microbial counts. After screening different LAB, statistically significantly (*p* < 0.05) higher microbial counts were found in 12 strains as a consequence of cava lees supplementation. Moreover, a greater and faster reduction in pH was observed in most of these strains. The growth-promoting effects of cava lees on LAB strains supports the potential revalorization of this winery by-product, either to improve the safety of fermented products or as a health-promoting prebiotic that may be selectively fermented by probiotic species.

## 1. Introduction

The production of cava, a highly appreciated Spanish sparkling wine, traditionally involves a second fermentation in the bottle and a subsequent biological aging of the base wine on lees for at least 9 months [1]. Cava lees are non-viable cells of *Saccharomyces cerevisiae* naturally plasmolyzed due to the sustained contact with wine [2,3,4]. In general, approximately one gram of lees, containing approximately 10^8^ dead cells of *S. cerevisiae*, is obtained for each bottle of cava [5]. Thus, considering that in 2020 around 215 million bottles of cava were produced in Spain, it can be estimated that about 215 tons of lees are generated annually [6]. These lees are currently considered a by-product of the cava industry that, according to its compositional characteristics, could potentially achieve a high added value.

Cava lees are rich in antioxidant compounds, as well as in soluble and insoluble fibers mainly derived from the yeast cell wall [7]. The principle components of the *S. cerevisiae* cell wall are β-1,3-glucan (50–55%) and β-1,6-glucan (5–10%), together with mannoproteins (35–40%), and chitin (1–2%) [8,9]. In recent years, β-glucans have attracted growing interest as an ingredient in food formulations [10,11] and, in 2011, β-glucans from yeast and fungi were approved by the European Commission as novel food ingredients [12,13]. Several studies have demonstrated the ability of β-glucans to promote the growth and survival of lactic acid bacteria (LAB) [14,15,16,17]. Similarly, mannan-oligosaccharides (MOS) have the capacity to stimulate the growth of different beneficial lactobacilli species of the intestinal microbiota, both in humans and animals [18,19,20].

The technological functionality of dietary fiber is increasingly being explored for food applications, especially in fermented products [21,22,23,24,25,26,27,28,29,30,31]. The addition of fiber can enhance the fermentation rate and help to achieve a greater decrease in pH, a desirable effect in terms of food safety. In this context, food industry by-products, reused as ingredients in the formulation of fermented meat products, have been found to stimulate the growth of certain LAB and provide safety advantages while maintaining or even improving the organoleptic quality of these foods [32,33].

From a health point of view, soluble fiber is well known to have a prebiotic effect that improves the survival and implantation of species within the intestinal microbiota with beneficial effects for the host [34]. Promising results have been recently reported about the potential prebiotic effect of fiber-rich by-products on the viability of certain probiotic strains, mainly lactobacilli. Thus, different tested probiotic strains showed an enhanced growth when cultured in vitro with a variety of food by-products, such as lemon peel, sugar beef pulp, horse chestnut seeds, fruit and vegetable sours, and apple pomace, although the growth-promoting effect has been described as strain-dependent [35,36,37,38]. 

Considering cava lees are rich in different types of fibers (mainly β-glucans and MOS), they have potential application as a microbial growth promoter, with benefits for food safety and health. If confirmed, this could be a novel strategy for the revalorization of this winery by-product in alignment with current circular economy initiatives. To date, cava lees have not been studied from this perspective. Therefore, the aim of this study was to assess in vitro the potential promoting effect of cava lees on the growth of different strains of LAB from species commonly used as starter cultures and/or probiotics. 

## 2. Materials and Methods

### 2.1. Obtaining Cava Lees

Cava lees were provided by the winery Freixenet S.A. (Sant Sadurní d’Anoia, Spain). To eliminate remaining cava, wet lees were centrifuged at 10,000 rpm for 10 min at 4 °C. The lees were subsequently frozen in an ultra-low temperature freezer (−80 °C), freeze-dried (Cryodos-50, Telstar, Terrassa, Spain), ground, and preserved in sealed tubes protected from the light and humidity until use.

### 2.2. Assessment of the Effect of Different Amounts of Cava Lees on the Growth of LAB Strains

**Figure 1:**
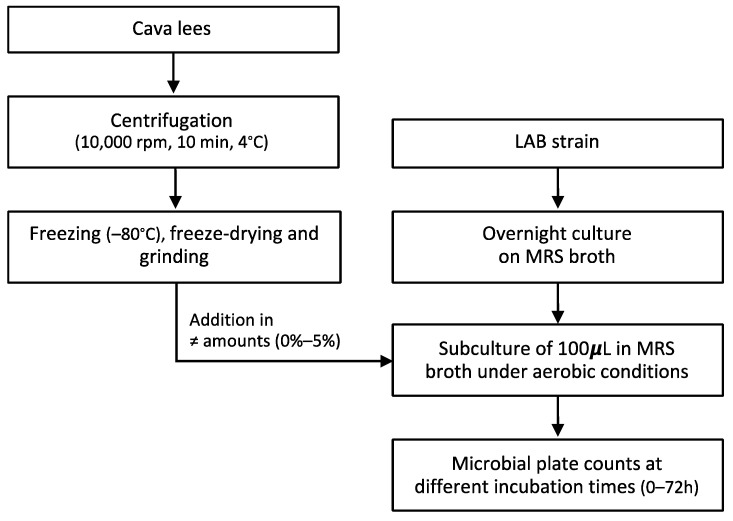
Overall experimental procedure for the in vitro assessment of the effect of cava lees on the growth of different LAB strains.

A scheme of the overall experimental procedure is provided in Figure 1. In order to select the most effective concentration, the growth promoter effect of different amounts of cava lees was assessed in 6 bacterial strains of *Latilactobacillus sakei* (CTC494 and BAP111), *Lactiplantibacillus plantarum subsp. plantarum* (CECT4180 and CECT220), *Latilactobacillus curvatus* (BAP202), and *Lacticaseibacillus casei* (BAP341). An overnight culture of each strain was performed in MRS broth (Condalab^®^, Madrid, Spain) incubated in optimal growth conditions (Table 1). Then, a 100 µL aliquot from the overnight culture was subcultured in 25 mL of MRS broth without (control) or with increasing amounts of lees (0.5%, 1%, 2%, and 5% (*w*/*v*)) at the optimal temperature, and with constant shaking (200 rpm) under aerobic conditions. Amounts above 5% of lees were not considered, as they technically hindered the cultivation of the bacterial strains. Plate microbial counts, expressed as log_10_ CFU/mL, were determined in duplicate at incubation times 0 to 72 h. Three independent assays were performed for each bacterial strain.

### 2.3. Screening of the Effect of Cava Lees on the Growth of LAB Strains

A total of 21 strains of LAB species were selected for their special interest from a technological (starter cultures) and health (probiotics) perspective, and all of them are included in the list of microorganisms with QPS (qualified presumption of safety) status (Table 1). The effect of the addition of 5% of cava lees (previously selected for showing the highest growth promoter effect) on each strain was tested as described in the previous section (Figure 1). Three independent assays were performed for each bacterial strain.

Changes in the pH of the culture media during the assay were measured by a digital instrument (pH 60 DHS^®^ ©XS instruments, Carpi, Italy). Due to the acidity conferred by lees, the pH value of the culture media supplemented with lees was adjusted with KOH (1M) to equilibrate the pH to that of the control sample.

### 2.4. Data Analysis

Statistical analysis of data was performed using the IBM SPSS Statistics 23.0 statistical software package (IBM Corporation, Armonk, NY, USA). Data of the growth promoter effect of different amounts of cava lees were assessed by an ANOVA and post hoc Tukey tests. Student’s *t*-test was applied to assess the statistical significance of log_10_ counts and pH values of each LAB strain cultured with and without lees. Differences with *p* < 0.05 were considered statistically significant.

## 3. Results and Discussion

### 3.1. Assessment of the Effect of Different Amounts of Cava Lees on the Growth of LAB Strains

**Figure 2:**
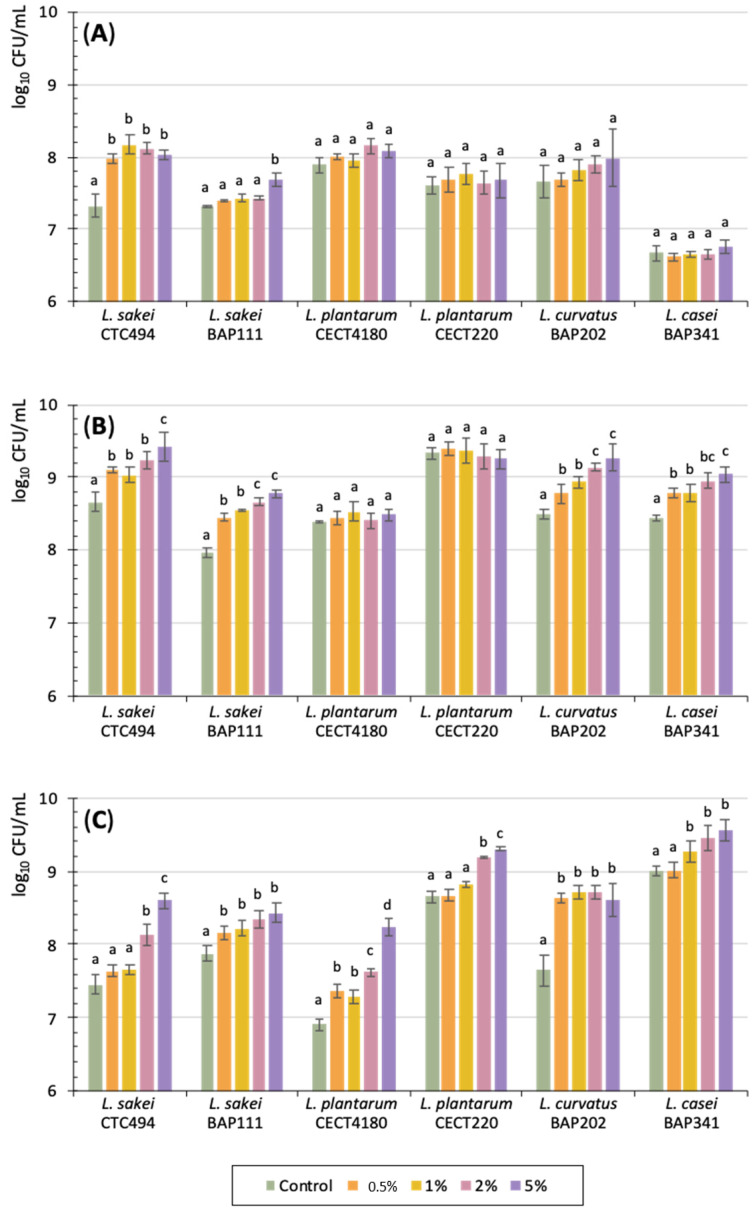
Concentration of 6 LAB strains in growth media without (control) and with different amounts of cava lees after 6 h (**A**), 24 h (**B**), and 48 h (**C**) of incubation. The average and standard deviation (bars) of three independent experiments are displayed. Different letters denote statistically significant differences (*p* < 0.05) between amounts of added cava lees.

Supplementation of the growth medium with cava lees resulted in a higher concentration of viable cells of the different LAB strains in comparison with the control medium (without lees). Overall, a strain-dependent and a dose-dependent effect was observed. As can be seen in Figure 2, the higher microbial counts of some strains due to cava lees was already observed after 6 h of culture, although statistically significant differences at this time were only obtained for *L. sakei* strains (*p* < 0.05). This effect was much more evident after 24 h, when the counts were significantly higher (*p* < 0.05) for four strains (*L. sakei* CTC494, *L. sakei* BAP111, *L. curvatus* BAP202, and *L. casei* BAP341) at all tested amounts of cava lees. The effect of cava lees differed according to the amount used, 2% and 5% producing the strongest statistically significant effect (*p* < 0.05) on bacterial growth (24 h) and/or survival after the stationary phase (at 48 h). Regarding *L. plantarum* strains, significantly different results associated with the concentration were not found until 48 h of incubation (*p* < 0.05). The highest microbial counts of *L. plantarum* were also obtained with 2% and 5% of lees, indicating that, at lower amounts or control conditions, microbial survival after the stationary growth phase was lower. Therefore, among the different assessed amounts of cava lees, the addition of 5% proved to be the most effective for all the tested strains, resulting in LAB microbial concentrations 0.6 to 1.3 log_10_ units higher than in the control cultures, either at 24 h or 48 h of incubation. Other studies have also reported a stimulatory effect on LAB growth of different plant-derived by-products at concentrations ranging from 1 to 10% [25,35,37,39,40]. Higher amounts (up to 20%) of purified oligosaccharides from soy sauce have also been described as beneficial for the growth of *Lactobacillus delbrueckii subsp. Bulgaricus* (previously *Lactobacillus bulgaricus*) and *Streptococcus thermophilus* after 48 h of supplemented culture [41].

### 3.2. Screening of the Effect of Cava Lees on the Growth of LAB Strains

**Figure 3:**
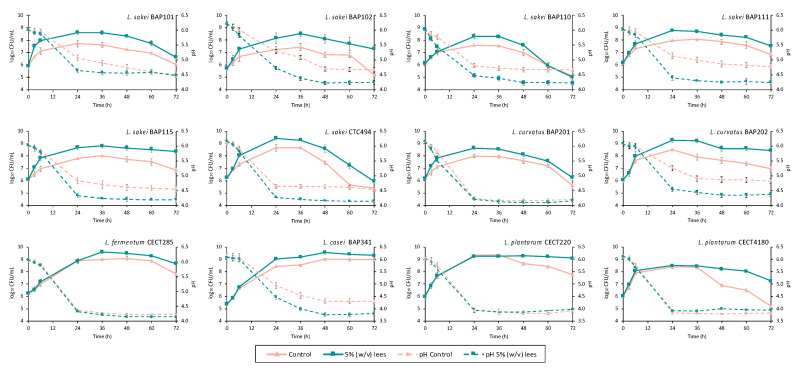
Growth kinetics and changes of pH values of LAB strains cultured without (control) and with 5% cava lees. The average and standard deviation of three independent experiments are shown. The enhanced bacterial growth in *L. sakei* strains cultured with cava lees also resulted in a greater decrease in pH in all strains (Figure 3). The increase in acidity of the culture media was significantly higher and faster during the first 24 h (pH values of 4.2–4.7, depending on the strain), coinciding with the exponential growth phase (*p* < 0.05). The pH of the control culture medium (without cava lees) was always higher, with values of 4.7–5.3.

The effect, in terms of growth, of adding 5% (*w*/*v*) of cava lees to the 21 tested LAB strains is shown in Table 1. A statistically significantly higher microbial count due to the presence of cava lees was observed at some time point in 12 strains; their growth kinetics and the pH decrease are depicted in Figure 3. The only species that did not show this effect were *L. gasseri* and *P. pentosaceus*. 

Overall, the higher microbial counts due to the addition of lees varied according to the strain (Figure 3). Regarding *L. sakei*, six out of eight strains showed a higher growth rate compared to the control (during the exponential growth phase in the first hours of incubation) when 5% of cava lees was added. Thus, significantly higher microbial counts were obtained at the end of the exponential phase for *L. sakei* BAP101, BAP102, BAP110, BAP111, BAP115, and CTC494, with differences in the maximum population density ranging from 0.7 to 1.1 log_10_ units with respect to the control (*p* < 0.05). Higher counts were generally maintained until the end of the 72 h incubation period (death phase). 

Regarding *L. curvatus*, a significant promotion of growth was observed for both tested strains compared with the control (*p* < 0.05). *L. curvatus* BAP202, whose growth rate was not affected in the first hours of incubation, reached a higher maximum population density, with concentrations up to 1.5 log_10_ greater than the control, and a pH 0.7 points lower. In the case of *L. curvatus* BAP201, despite an enhanced growth in the presence of cava lees, the level of acidification was very similar with or without the addition of lees. 

*L. fermentum* CECT275 and *L. casei* BAP341 were also influenced by the addition of lees, although to a lesser extent than the *L. sakei* and *L. curvatus* strains (Figure 3). Significant differences in maximum population density and/or survival of both bacterial strains appeared after 24 h of culture, with increases generally higher than 0.5 log_10_ cfu/mL compared to the control (*p* < 0.05). However, a significant effect on acidity was only observed for *L. casei* BAP341, the final pH value being 0.5 units lower compared to the control (*p* < 0.05). 

*L. plantarum* were the bacterial species least affected by the presence of cava lees. Of the six strains tested, only two (*L. plantarum* CECT220 and *L. plantarum* CECT4180) showed any differences with respect to the control, though only regarding the survival on the last day of the incubation period. In fact, the addition of cava lees to the culture media significantly enhanced the viability after 36 h (*p* < 0.05), *L. plantarum* CECT220 (1.4 log_10_) and CECT4180 (2 log_10_), showing a higher concentration compared to the control culture. No differences were observed in the pH values between the supplemented and unsupplemented culture media, which could be explained by the lack of effect of cava lees in the growth phase in both strains.

Various plant-based by-products have been successfully applied to improve the nutritional, textural and sensory properties of dairy and meat fermented products [24,25,28,29]. However, there are few data about the enhancing effect of by-products on the microbiological safety of fermented products [32,33]. Sayas-Barberá et al. [32] demonstrated a positive effect of a 1% fiber-rich by-product of orange juice production on the food safety of dry fermented sausages when combined with the probiotic strain *L. casei* CTC475. Supplementation with this citrus by-product promoted the growth and survival of LAB, resulting in a higher acidification of sausages without affecting their sensory properties. More recently, the effect of sake lees (i.e., the residue of the brewing process of the Japanese fermented rice beverage) on dry-fermented sausages was assessed, and higher LAB counts and lower pH values during the aging period were observed [33].

Several recent studies have focused on the revalorization of by-products with a potential role as prebiotics [35,36,37,38,39,40,42]. Przyborska et al. [36] reported the prebiotic capacity of a horse chestnut by-product, which induced a higher in vitro growth of various probiotic strains of *L. plantarum*, *L. casei* and *L. fermentum*, among others. As in the current study, the capacity to ferment this substrate varied among strains. Andrade et al. [37] found an increased growth of four probiotic lactobacilli strains cultured in vitro with 3% of fruit and vegetable flour. Later, the prebiotic effect of this fiber-rich by-product was further confirmed on gut microbiota obtained from fecal samples. Similar results were obtained by Massa et al. [39] regarding the in vitro prebiotic effect of peel and seeds from jabuticaba, a Brazilian fruit. 

The bacterial growth-promoting effects of the above-mentioned by-products have been mainly attributed to their content of oligo- or polysaccharides (e.g., β-glucans, arabinans, arabinogalactans, galactans, xyloglucans, and galactomannans) and pectic substances. Likewise, the observed growth enhancement induced by cava lees could be due to the bacterial use of β-glucans and MOS (constituting more than 90% of the cell wall) as a carbon source. In this regard, the capacity of β-glucans (usually obtained from microbial cell walls, oat, or rye) and MOS to stimulate the growth of several lactobacilli has been extensively reported [15,16,23,27,43,44,45,46,47].

The metabolism of these large carbohydrates requires, as a first step, their hydrolysis into fermentable smaller sugars by glycosidases [15]. The presence of these enzymes has been reported in the *Lactobacillus* genus, which may endow these bacteria with the ability to use oligo- or polysaccharides as a carbon source [15,48]. This is consistent with the results of a study by Russo et al. [15], in which β-glucans showed higher prebiotic activity on a recombinant *L. plantarum* strain with an overexpressed B-glycosidase enzyme. However, the enzymes responsible for this metabolic pathway may be disabled in certain bacterial strains, thus compromising their ability to ferment these substrates [49,50,51]. This fact supports the results obtained herein, according to which the supplementation with cava lees showed a strain-dependent behavior on the growth-promoting effect of technological and probiotic LAB strains. 

Overall, the enhancement of LAB strain growth in vitro and reduction in pH values induced by cava lees supports the potential revalorization of this winery by-product with applications to improve the safety of fermented foods. Furthermore, the selective fermentation of cava lees by certain probiotic species suggests they could be exploited as prebiotics in health applications. Higher amounts of cava lees could potentially have a growth-promoting effect on those species in which no in vitro effect has been observed. Thus, the results obtained here need to be confirmed in real models of fermented foods, which would also allow us to assess both the effects of higher amounts of cava lees and the impact on organoleptic properties. It should be noted that the observed strain-dependency of the effects of cava lees warrants case-by-case study. The proposed recycling of cava lees represents a novel strategy for the revalorization of a by-product generated in large amounts. This approach is in alignment with the Sustainable Development Goals of the United Nations, focused on reducing food industry waste products and promoting the circular economy.

## Figures and Tables

**Table 1:** Lactic acid bacteria (LAB) strains used in this study.

Species	Strain	Optimal Growth Temperature	Source (Collection)	Growth Promoted by Cava Lees
*Latilactobacillus sakei*	BAP101	30 °C	Research Group Collection	YES
	BAP102			YES
	BAP110			YES
	BAP111			YES
	BAP115			YES
	CTC494		IRTA culture collection ^1^	YES
	SM181		CHR Hansen^®^	NO
	FSC111			NO
*Latilactobacillus curvatus*	BAP201	30 °C	Research Group Collection	YES
	BAP202			YES
*Lactiplantibacillus plantarum subsp. plantarum*	CECT4180	37 °C	Spanish Type Culture Collection	YES
	CECT220			YES
	CECT221			NO
	CECT224			NO
	CECT749			NO
	ATCC14917	37 °C	American Type Culture Collection	NO
*Lacticaseibacillus* *casei*	CECT7600	37 °C	Spanish Type Culture Collection	NO
	BAP341		Research Group Collection	YES
*Limosilactobacillus fermentum*	CECT285	37 °C	Spanish Type Culture Collection	YES
*Lactobacillus gasseri*	BAP513	37 °C	Research Group Collection	NO
*Pediococcus pentosaceus*	CECT8330	37 °C	Spanish Type Culture Collection	NO

^1^ Institute of Agrifood Research and Technology.
